# Spinal radiosurgery - efficacy and safety after prior conventional radiotherapy

**DOI:** 10.1186/1748-717X-6-173

**Published:** 2011-12-16

**Authors:** Katharina Nikolajek, Markus Kufeld, Alexander Muacevic, Berndt Wowra, Maximilian Niyazi, Ute Ganswindt

**Affiliations:** 1 Department of Radiation Oncology, Ludwig-Maximilians-University, Campus Grosshadern, Marchioninistr. 15, 81377 München, Germany; 2 Cyberknife Center, Max-Lebsche-Platz 31, 81377 München, Germany

## Abstract

**Background:**

Conventional external beam radiotherapy is a standard procedure for treatment of spinal metastases. In case of progression spinal cord tolerance limits further radiotherapy in pre-irradiated areas. Spinal stereotactic radiotherapy is a non-invasive option to re-treat pre-irradiated patients. Nevertheless, spinal radiosurgery results in relevant dose deposition within the myelon with potential toxicity. Aim of the study was to retrospectively analyse the efficacy and feasibility for salvage radiosurgery of spinal metastases.

**Methods:**

During a period of 4 years (2005-2009) 70 lesions in 54 patients were treated in 60 radiosurgery sessions and retrospectively analysed. Clinical (pain, sensory and motor deficit) and radiological (CT/MRI) follow-up data were collected prospectively after radiosurgery. Pain - as main symptom - was classified by the Visual Analogue Scale (VAS) score. Every patient received single session radiosurgery after having been treated first-line with conventionally fractionated radiotherapy. Kaplan-Meier method and life tables were used to analyse freedom from local failure and overall survival.

**Results:**

At a median follow-up of 14.5 months the actuarial rates of freedom from local failure at 6/12/18 months were 93%, 88% and 85%, respectively. The median radiosurgery dose was 1 × 18 Gy (range 10-28 Gy) to the median 70% isodose. The VAS score of patients with pain (median 6) dropped significantly (median 4, p = 0.002). In 6 out of 7 patients worse sensory or motor deficit after SRS was caused by local or distant failures (diagnosed by CT/MRI). One patient with metastatic renal cell carcinoma developed a progressive complete paraparesis one year after the last treatment at lumbar level L3. Due to multiple surgery and radiosurgery treatments at the lumbar region and further local progression, the exact reason remained unclear. Apart from that, no CTC grade III or higher toxicity has been observed.

**Conclusions:**

By applying spinal radiosurgery relevant radiation doses can be limited to small parts of the myelon. This prevents myelopathic side effects and makes it an effective and safe treatment option for well-suited patients. Especially for previously irradiated patients with local failure or pain salvage SRS represents a valuable treatment option with high local control rates, low toxicity and significant pain reduction.

## Background

**Figure 1:**
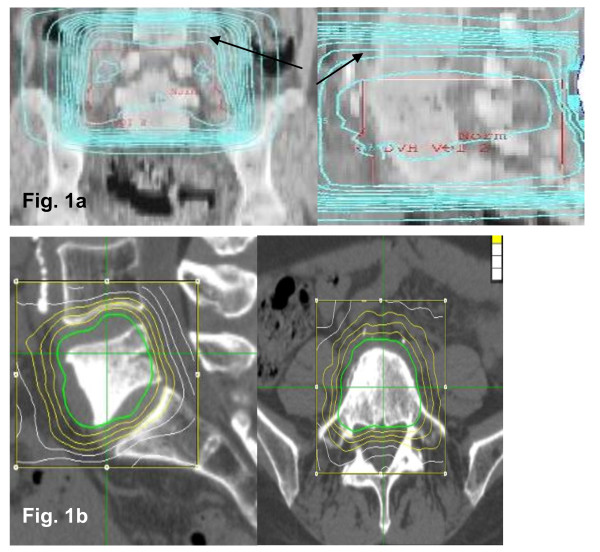
**3-D planning in a patient with osseous metastasis in the lumbar vertebral body 5**. PTV for conventional radiotherapy (Figure 1a) included L4-L1 (**→ **= encompassing 95%-isodose). Below (Figure 1b) salvage SRS planning in the same patient at lumbar vertebral body 5 after conventional radiotherapy (green: 70%-isodose, yellow: 60%/50%/40%-isodose, white: 30%/20%-isodose). Abbreviation: SRS = spinal radiosurgery.

Conventional radiotherapy (Figure 1a) is an evidence-based treatment to control pain, neurological symptoms and instability of spinal metastases (in general osseous metastases, partially expanded to the spinal canal, [1] or other spinal tumours). Rades et al. [2] described an in-field recurrence rate up to 26% for patients with spinal metastases treated with short course radiotherapy (1 × 8 Gy or 5 × 4 Gy). Patients treated with long course radiotherapy (10 × 3 Gy, 15 × 2.5 Gy or 20 × 2 Gy) showed a significantly better local control.

Stereotactic radiotherapy using either single fraction treatments ("radiosurgery", Figure 1b) or hypo-fractionated approaches is an established treatment option for a variety of malignant and non-malignant conditions including lung cancer, metastases and CNS tumours [3-9]. The development of modern techniques including intensity modulated radiotherapy (IMRT), small multi-leaf collimators (MLC), precise fixation systems and optimized dose calculations, increase the accuracy and fidelity of radiosurgery and stereotactic radiotherapy [10-14]. In most situations stereotactic radiotherapy is used for either small volume lesions, small volume recurrences after prior radiotherapy or as boost-radiotherapy in the initial management of tumours [8,9,15].

In this regard, spinal radiosurgery (SRS) has proved to be an important and convenient option in the treatment of spinal metastases for properly selected patients [16-19]. For example, SRS is not recommended in patients with a spinal cord canal compromise > 25%, significant or progressive neurological deficit or spine instability [20]. Moreover the current ASTRO guidelines for the palliative treatment of bone metastases [21] clearly recommend conventional radiotherapy primarily for the treatment of pain and/or prevention of the morbidity caused by bone metastases.

In case of progression or local relapse spinal cord tolerance often limits further radiotherapy in previously irradiated areas. For patients without surgical options SRS offers a possibility to treat previously irradiated regions. Stereotactic radiotherapy of the spine reduces the radiation exposure to the spinal cord and therefore potential neurological deficiency. The aim of this study was to retrospectively analyse the clinical outcome for radiosurgery of spinal metastases in previously irradiated patients.

## Methods

During a period of 4 years (2005-2009) 70 lesions were treated with SRS (CyberKnife^®^) in 54 patients (32 male, 22 female). All patients had either clinical (pain or neurological) symptoms or radiological (CT/MRI) progress in the area or in the boundary area of the previously irradiated fields.

The pre-irradiation treatment parameters were documented systematically (date, target volume, daily dose, total dose, dose to the spinal cord and CT-planning data as far as available).

For SRS treatment CyberKnife^® ^system and the skeletal structure tracking software (Xsight) were used. Patients received planning CT-scans in supine position with 1.0 mm slice thickness. MRI scans were fused with the planning CT images to augment soft tissue discrimination. The planning target volume (PTV) was generated using the gross tumour volume (GTV) without any intended margin. However, a certain margin resulted since the surrounding isodose was calculated to extend maximal 5 mm towards the vertebral body and 0-2 mm towards the spinal cord (Figure 1b). In some cases PET-scans were employed to differentiate between necrotic and relapsed tumour.

The selected SRS single fraction dose depended on actual tumour size, the proximity of the tumour to critical structures - especially the myelon - and previous irradiation dose and fields. The generally intended prescription dose was 20 Gy calculated to the 70% isodose. However, as mentioned above, depending on either some risk factors or characteristics of PTV coverage this dose prescription was adapted individually. Moreover, the maximum dose to the spinal cord was basically intended not to exceed 8 Gy.

For treatment delivery the patients were freely placed on the treatment couch in the same position as during planning CT-scan. The bone position was verified by orthogonal X-ray images. Analgesic or sedative medication was given if required.

The radiological follow up was performed with CT or MRI imaging at 3-months-intervals and documented prospectively. Using the RECIST criteria "stable disease" and "partial or complete remission" were defined as local control, whereas "progressive disease" was defined as local failure. Additional clinical follow up at 3-months-intervals included neurological examination and a standardized asking procedure for pain. Pain as a main symptom was classified by using the VAS score. Apart from this prospective evaluation of clinical outcome data the irradiation treatment parameters of both conventional and stereotactic radiotherapy were collected and analysed retrospectively.

### Statistical Analysis

The Stata/IC 10.1 software (Stata Corp., College Station, Texas, USA) was used for statistical analysis. Kaplan-Meier method and life tables were calculated to analyse overall survival and freedom from local failure. We used the log-rank test to analyse the outcome for patient characteristics (sex, age, Karnofsky performance status scale), tumour characteristics (primary tumour, quantity of treated spinal lesions) and treatment-related variables (single/total dose of conventional pre-irradiation, radiosurgery dose, > 8-Gy-volume to the dural sac, time interval conventional radiotherapy to SRS). We used the t-test to analyse VAS score as a classification for pain.

## Results

70 lesions in 54 patients (32 male, 22 female) with different primary tumour sites (sarcoma, CNS, chordoma, renal, lung, breast, prostate, colorectal, gynaecological, melanoma and others) were treated in 60 radiosurgery sessions. Out of these 54 patients, 13 had progressive disease at the primary spinal/paraspinal tumour site, whereas 41 patients suffered from metastatic disease. Within the 41 metastatic patients most common lesions were caused by renal cell carcinoma (n = 10; 18.5%) and non-small-cell lung cancer (n = 7; 13%). 14 lesions in 13 patients were located intraspinal/-dural, while 56 lesions represented osseous or paraspinal lesions. In addition, 26 patients had undergone at least secondary surgery and 30 patients had received chemotherapy before radiosurgical treatment.

All spinal regions, but most frequently thoracic and lumbar lesions, were affected. Detailed patient and characteristics are shown in table 1.

**Table 1:** Patient, tumour and treatment characteristics for all patients

Characteristic	Value	
Median age (years), (range)	56, (17-82)	

No of patients	54	

Sex		
Male	32	
Female	22	

Karnofsky performance status scale		
Median	80	
Range	50-100	

Spinal region/No. of treated lesions		(%)
Total of lesions in 54 patients	70	(100)
Patients with 1 lesion	43	
Patients with 2-6 lesions	11	
Cervical	8	(11.4)
Thoracic	28	(40.0)
Lumbar	27	(38.6)
Sacral	7	(10.0)
Total of treatment sessions	60	
No. of treated lesions/treatment sessions		
1	42	
2	9	
3	2	
4	1	

Histology	n	(%)
Primary tumours	13	(24.1)
Sarcoma	5	(9.3)
CNS	3	(5.6)
Chordoma	3	(5.6)
Others	2	(3.7)
Metastases	41	(75.9)
Renal	10	(18.5)
Lung	7	(13)
Breast	7	(13)
Prostate	3	(5.6)
Colorectal	2	(3.7)
Gynaecological	2	(3.7)
Melanoma	1	(1.8)
Others	7	(13)

Time diagnose to SRS (years)		
Median	4.6	
Mean	6.7	
Range	0.6-29	
95% C.I.	2.4-6.4	

Time RTX to SRS (months)		
Median	15	
Mean	42	
Range	1-505	
95% C.I.	13.8-26.9	

Tumour volume - GTV (cc)		
Median	17.6	
Mean	26.3	
Range	0.2-134	
95% C.I.	12.2-26.2	

Abbreviations: SRS = spinal radiosurgery; RTX = conventional radiotherapy.

All 70 lesions were located in conventionally pre-irradiated volumes. In 47 patients the prior conventional radiotherapy (range of daily doses 1.6 - 3 Gy, 1 patient 1 × 8 Gy) decidedly covered parts of the vertebral column/spinal cord (but also 1 patient with whole body irradiation, 2 patients with irradiation of entire craniospinal axis, all other patients ≥ 3 vertebral bodies), whereas 7 patients had been treated in the course of curative intended 3D-plannings (mediastinal, paraaortic, paraspinal region) with partial dose deposition to the spinal cord (single doses 1.8 - 2 Gy). Based on the available documents of prior radiotherapy, we re-calculated the maximum total dose applied to the myelon for the whole cohort using the linear-quadratic model (α/β = 2, daily dose 2 Gy). Thus the median re-calculated nBED Gy 2/2 to the spinal cord was 42.8 Gy (mean 41.5 Gy, range 18 - 48.7 Gy).

32 of 54 patients suffered from significant pain before SRS (median VAS score 6). 14 patients suffered from complete or incomplete paresis, 12 patients suffered from par-/hypaesthesia and 5 patients had both motor and sensory deficits before SRS treatment.

The median time to SRS re-treatment was 15 months (95% C.I. 14-27 months).

The median encompassing dose applied by SRS was 18 Gy (range 10-28 Gy) to the median 70%-isodose. Additionally the median tumour volume was 17.6 cc (mean 26.3 cc, range 0.2-134 cc), the median > 8-Gy-volume to the dural sac was 0.74 cc (mean 1.4 cc, range 0-9 cc). With the exception of two patients, who were treated with re-radiosurgery at the same lumbar level (L3, but contralateral vertebral body involvement) all metachronously treated lesions were distant. Detailed SRS treatment parameters are shown in table 2.

**Table 2:** SRS treatment parameters

Parameter	Value
Prescription dose (Gy) 70% isodose	
Median	18
Mean	18.1
Range	10-28
95% C.I.	17-18

Dose maximum (GTV)	
Median	25.7
Mean	26.8
Range	20-43
95% C.I.	23.9-28.6

Dose minimum (GTV)	
Median	12.2
Mean	12
Range	3.8-20.3
95% C.I.	11.4-12.7

Peripheral isodose (%)	
Median	70
Mean	67
Range	50-80
95% C.I.	65-70

No. of beams/site or lesion	
Median	215
Mean	207
Range	64-341
95% C.I.	182-239

10 Gy irradiated volume (cc)	
Median	95
Mean	120
Range	0.6-479
95% C.I.	63-132

> 8 Gy volume of spinal cord (cc)	
Median	0.74
Mean	1.4
Range	0-9.3
95% C.I.	0.4-1.2

**Figure 2:**
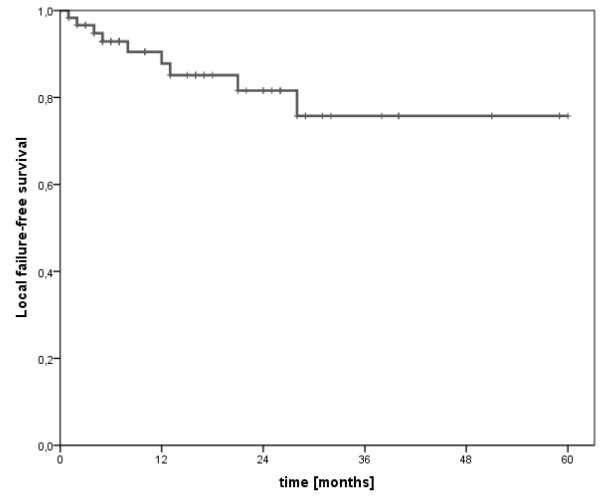
**Local failure-free survival after salvage spinal radiosurgery**.

At a median follow up of 14.5 months (range 3-48 months) we observed 9 (12.9%) local failures (7 "in-field" and 2 "field-margin" relapses, 10% and 2.9%, respectively) within the 70 treated lesions. The actuarial rates of freedom from local failure at 6/12/18 months were 93%/88%/85% (Figure 2). As a result in the subgroup of patients with local failure after SRS we observed significant larger tumour volumes treated by SRS (p = 0.001). Moreover, the median time interval from conventional radiotherapy to SRS displayed a trend to be shorter in this subgroup (p = 0.165). Detailed tumour and treatment characteristics of patients with local failure are shown in table 3.

**Table 3:** Tumour and treatment characteristics for patients with local failure (n = 9)

**No**.	Tumour type	Location	Volume (cc)	Prescription Dose (Gy) SRS	Isodose (%) SRS	Time (months) RTX to SRS	Prior RTX Dose nBED Gy 2/2
1	RCC	T11	93.3	16	65	21.7	42,8

2	RCC	L3	134.2	19	55	8.8	40

3	Chordoma	L2	39.7	22	65	12.4	40

4	Prostate	T11	89.8	18	65	2.8	45

5	Melanoma	C7	17.7	17	65	4.3	33.6

6	Sarcoma	L4	56.3	20	70	2	44

7	Breast	L3	37.6	20	70	28.8	42.8

8	MPNST	T1	26.8	14.5	70	5.8	42.8

9	RCC	T9	49.9	22	70	13.9	42.8

	Median	-	49.9	19	65	8.8	42.8

	Mean	-	60.6	18.8	66	11.2	41.6

	Range	-	18-134	14.5-22	55-70	2-28.8	33.6-45

Abbreviations: SRS = spinal radiosurgery; RTX = conventional radiotherapy; RCC = renal cell carcinoma; MPNST = malignant peripheral nerve sheath tumour

**Figure 3:**
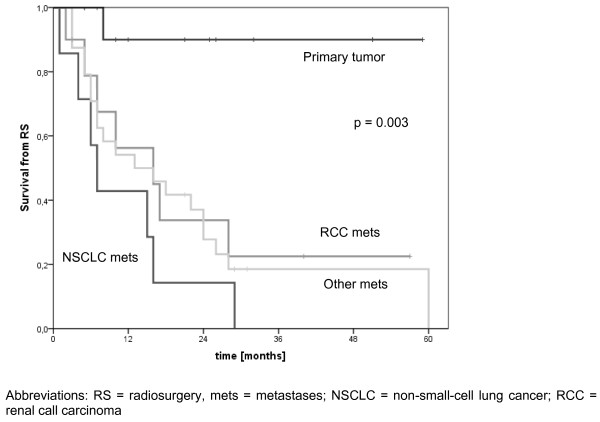
**Overall survival from salvage spinal radiosurgery of most common tumour sites**. Abbreviations: RS = radiosurgery, mets = metastases; NSCLC = non-small-cell lung cancer; RCC = renal call carcinoma.

For the whole cohort the median overall survival after SRS treatment were 16.2 months and 42 months after initial radiotherapy, respectively. Patients being re-irradiated with SRS at their initial primary tumour region had a significant better overall survival when compared with patients suffering from metastatic tumour disease (p = 0.003, Figure 3).

Among the patients with initial pain symptoms the VAS score dropped significantly within 3 months after the treatment (median VAS score 4, p = 0.002). After SRS in 7 patients impairment of neurological symptoms was found (table 4). The exact assignment of neurological symptoms to the spinal level was not always clearly possible. One patient with renal cell carcinoma developed progressive paraparesis one year after the last treatment of a widespread spinal metastasis at lumbar level L3 (laminectomy with internal stabilization, conventional radiotherapy, 2 times stereotactic radiosurgery at different locations of L3, metachronous SRS at L2 and L4, surgery for recurrence). Due to the multiple treatments and further tumour progression in this area, the exact reason for paraparesis could not be identified. However, radiation induced myelopathy has to be considered due to the frequent radiosurgery treatments at different lumbar levels (patient with maximum of lesions/lesion volumes and treatment sessions in our cohort).

**Table 4:** Clinical (pain/neurological) outcome of all treatment sessions

Outcome parameter	Value
Total No. of treatment sessions	60

Pain	
VAS Score median pre-RS (n = 60)	2
VAS Score median post-RS (n = 60)	0
p-value pre-/post-RS (n = 60)	0.07
Patients without initial pain pre-RS	26
VAS Score median pre-RS	0
Patients with pain pre-RS	32
VAS Score median pre-RS	6
VAS Score median post-RS	4
p-value pre-/post-RS	0.0056

Sensory deficit	
No. of patients pre-RS	12
Sensory deficit post-RS	
Better	1
Same	9
Worse	2

Motor deficit	
No. of patients pre-RS	14
Motor deficit post-RS	
Better	1
Same	8
Worse	5

Apart from that, no CTC grade III or higher grade neurological or other (necrosis, bleeding) toxicities have been observed.

## Discussion

Aim of our study was to analyse the clinical outcome of salvage radiosurgery for spinal tumours/metastases in previously irradiated patients in regard to efficacy (local control, pain relief, neurological symptoms) and safety (potential side effects). At a median follow up of 14.5 months our results in 54 patients with 70 treated lesions by a median SRS dose of 18 Gy (range 10-28 Gy) to the median 70%-isodose provide evidence that single-dose application is associated with high local control rates. In regard to symptoms of pain by using the VAS score prospectively we observed a significant decrease of pain in 75% of the patients. 7/54 patients developed an impairment of neurological (2/54 sensory and 5/54 motor) deficits after SRS, among them 6 patients with causally progressive local or distant failures.

By all means, stereotactic radiotherapy is an attractive way to treat spinal metastases non-invasively with encouraging results in selected patients. Although technical feasibility and growing expertise in stereotactic treatment approaches increased accuracy and fidelity of spinal SBRT and SRS [16,18-20,22-26] some important challenges on this topic still remain under discussion.

The QUANTEC data review published in 2010 estimated the risk for myelopathy < 1% and < 10% at 54 Gy and 61 Gy, respectively, when conventional fractionation (1.8-2 Gy) is used [27]. However, in the case of single-dose or hypo-fractionated radiotherapy the maximum spinal cord tolerance is quite more uncertain and sufficient long-term-data are missing [27]. Sahgal et al [28] calculated a dosimetric modelling by comparing SRS parameters in not pre-irradiated patients with myelopathy (n = 5) and patients without myelopathy (n = 19) after SRS, respectively. Based on these results they recommend a point dose threshold of 10 Gy for a single fraction (nBED of 30 Gy 2/2) to the thecal sac. Ryu et al. [29] described a partial volume tolerance to the spinal cord of 10 Gy to 10% of the spinal cord volume defined as 6 mm above and below the radiosurgery target. Gibbs et al. analysed 6 cases of delayed myelopathy after SRS: A total of 1075 patients treated with radiosurgery were investigated and 6 patients developed a delayed myelopathy. Specific dosimetric parameters associated with this complication could not be identified apart from the fact that 3 of the 6 analysed patients received spinal cord doses above 8 Gy [26].

Nevertheless, the problem of cumulative spinal cord tolerance for conventional radiotherapy followed by stereotactic radiotherapy is crucial. Some recent publications also showed efficacy and safety of salvage SRS or hypo-fractionated stereotactic radiotherapy (table 5).

**Table 5:** Studies on spinal stereotactic re-irradiation (SBRT/SRS) after conventional radiotherapy

Author	**Gerszten et al. 2007 **[16]	**Sahgal et al. 2009 **[30]	**Choi et al. 2010 **[31]	**Garg et al. 2011 **[25]
No of patients	344/500*	39*	42	59

No. of lesions	≈344	37/60*	51	63

Tumour volume (cc)				
Mean/median (range)	46/29 (0.2-264) (n = 500*)	-/21 (0.4-177) (n = 37)	-/10.3 (0.2-128.6)	67.1/51.2 (3.5-266)

SRS/SBRT	1 × 12.5-25 Gy (mean 20 Gy)	8-30 Gy (median 24 Gy)	10-30 Gy (median 20 Gy)	5 × 6 Gy
fractionation		1-5 fractions (median 3)	1-5 fractions (median 2)	3 × 9 Gy
				
Peripheral isodose	80%	60%		95%

Follow up (months)	21 (median) (n = 500*)	7 (mean)	7 (mean)	17.6 (mean)

Local control				
6 months			87%	
12 months		96% (n = 31)	73%	76%
"long term"	88% (n = 294*)			

Overall survival	-			
Median (months)		21		
6 months			81%	
12 months			68%	76%
24 months		45%		

Previous RT dose	10 × 3 Gy or 14 × 2.5 Gy	Median 36 Gy 14 fractions	Median 40 Gy 2 Gy dose equivalent	< 45 Gy (subgroup analysis </>35 Gy)

Abbreviations: SRS = radiosurgery; SBRT = Stereotactic body radiotherapy; *cohort included patients with/without pre-irradiation

Sahgal et al. [30] analysed 39 patients with 60 lesions, 37 of 60 lesions had previous irradiation. Thereby a dose of 24 Gy in three fractions to the 60%-isodose was used, the median ≥ 8 Gy volume to the spinal cord/cauda equina was 0.3 (range 0-28) cc and 0.3 (range 0-17) cc, respectively. No myelopathy or radiculopathy occurred. For the salvage group the 1-year progression-free probability was 96%, the median pre-irradiation total dose was 36 Gy/14 fractions. However, the median follow-up time of 7 months was rather short. There was no significant difference in regard to clinical outcomes between previously irradiated or un-irradiated patients. By comparison to our data with a median follow up of 14.5 months we observed a 1-year local control rate of 88% and the median ≥ 8 Gy volume was 0.74 (range 0-9) cc in our cohort. With regard to the pre-irradiation dose we documented a slightly higher median nBED Gy 2/2 (42.8, range 18 - 48.7 Gy) to the spinal cord.

Choi et al. [31] confirmed safety and performance for SRS after prior irradiation in patients with spinal metastases as well. They reviewed 51 lesions in 42 patients with a median previous spinal cord dose of 40 Gy. SRS was delivered to a median marginal dose of 20 Gy (range 10-30 Gy) in 1-5 fractions (median 2), according to a converted median equivalent single-session tumour dose of 15 Gy (α/β = 10). With a median follow-up of 7 months the local control rates at 6/12 months were 87%/73%. Median single-session equivalent spinal cord maximum doses (α/β = 3) were 10.9, 13.8, 12.5, 12.1, and 12.1 Gy for 1- to 5-session SRS. One patient (2%) experienced Grade 4 neurotoxicity; a significant pain relief was observed in 65% of the patients. Remarkably, the time to re-treatment (≤ 12 months) together with a single-session equivalent dose ≤ 15 Gy was associated with a significantly higher risk for local failure. In summary, they discuss an increased efficacy of higher single-doses in case of a short period of tumour recurrence assuming that the short time interval between irradiation and recurrence can be interpreted as more aggressive tumour growth behaviour. In this context our subgroup analysis of patients with local failure revealed a median time interval to re-treatment of 8.8 (range 2-28.8) months. However, we did not observe any differences concerning the applied SRS doses, but our results demonstrated significantly larger tumour volumes in this patient group. Bearing in mind that a longer time interval between two irradiation series to the spinal cord is assumed to be associated with a lower risk for severe late toxicity [27,32], both the predictive and prognostic factor "time to re-treatment" and "tumour aggressiveness" causally increases the risk of neurological complications in such patients.

Gerszten et al. reported the largest series of 500 cases treated with single-session SRS (mean single dose 20, range 12.5-25 Gy) including 344 pre-irradiated patients [16]. At a median follow up of 21 months the long-term local control was 88% for the most common primary tumour sites (294 pre-irradiated and non-irradiated patients, see table 5). Long-term pain improvement occurred in 290 of 336 (pre-irradiated and non-irradiated) cases. The mean < 8 Gy volume to the spinal canal was 0.6 cc for the whole cohort. Regarding the endpoints pain and local control the authors conclude that salvage SRS for metastases is both safe and clinically effective, especially for patients with solitary sites of spine involvement.

In 2011 Garg et al. reported a prospective evaluation of spinal re-irradiation by using SBRT (5 × 6 Gy and 3 × 9 Gy prescription dose, mean total dose constraint to the spinal cord 10 and 9 Gy, respectively) in 59 patients [25]. At a median follow up of 17.6 months they observed an actuarial 1-year local control rate and overall survival of both 76%. The CTC and McCormick neurological function system were used to evaluate toxicity and neurological status, respectively. Freedom from neurologic deterioration from any cause was 92% at 1 year. Two patients experienced mild to moderate radiation injury (lumbar plexopathy). Based on the performed local relapse analysis the authors conclude that initial surgery should be considered for tumours within 5 mm of the spinal cord.

Taken together, this data provide strong evidence that salvage stereotactic radiotherapy in pre-irradiated patients can be regarded as safe and effective treatment option. Despite some limitations presented data correlate well with those from literature, especially regarding the clinical outcome in terms of local control and pain relief. Some limitations particularly result from retrospective analysis of radiotherapy treatment parameters and there should be some caution in interpreting the data. Due to the fact that previous conventional radiotherapy often had been applied without the use of 3D planning, exact dose distributions were not available in all cases. Thus we did not calculate a cumulative nBED value for the individual patient. In this context the re-calculated median nBED Gy 2/2 of prior conventional radiotherapy (42.8 Gy) in our cohort was slightly higher than reported by other groups. Moreover, reported patient collective may be rather heterogeneous including 13 non-metastatic patients with progressive primary tumour at the initial high-dose pre-irradiated tumour site. Although overall survival rate in this patient subgroup was significantly better (Figure 3) the 1-year OS rate of 62% for the whole cohort is worse than reported by other series. In addition, both our percentage of intraspinal/-dural lesions (20%) in opposition to osseous or paraspinal lesions and 14/54 patients with motor deficit prior to SRS have to be borne in mind.

Beside highly important challenges in technical conditions accompanied by increasing accuracy and fidelity of spinal SBRT a precise patient selection in regard to clinical outcome is of special importance. Special criteria have to be fulfilled like singular, small tumour volumes. In not pre-irradiated patients the best benefit of treatment is expected in patients with good clinical condition and severe tumour associated pain. As mentioned above patients with spinal cord compression more than 25% with significant or progressive neurologic deficits or spine instability should not be treated with SRS [16-20,25]. However, in patients compromised by previous irradiation and missing surgical options SRS offers the only effective treatment option in some clinical constellations.

The question in how far SRS may replace in future any conventional radiotherapy in metastatic patients remains under debate. Patients with spinal metastases often suffer from pain, neurological deficiency or instability of vertebral bodies. Conventional radiotherapy has been proven to be an effective option in the treatment of spinal metastases [1]. A standard fractionation schedule for conventional radiotherapy depends on individual aspects of the patient like overall prognosis or clinical symptoms. Lower daily fractions are correlated with better remineralisation and longer pain relief than short courses with high single doses, so patients with oligometastases and a good prognosis may benefit from lower daily fractions [1,23,33]. Accordingly, Rose et al [34] described in a recent publication a higher risk of bone fracture after first-line treating spinal metastases located between T10 and os sacrum with high single dose intensity-modulated radiotherapy.

In this context the current ASTRO guidelines for the palliative treatment of bone metastases [21] clearly state that conventional radiotherapy continues to be the mainstay for the treatment of pain and/or prevention of the morbidity caused by bone metastases. The Task Force recommended that the use of SBRT/SRS in the primary treatment setting should be limited to highly selected patients and preferably within a prospective trial.

## Conclusions

Under certain technical conditions SRS is an effective and safe option to treat previously irradiated spinal metastases for properly selected patients. In cases of local failure salvage SRS reveals high local control rates with low toxicity and significant pain reduction. Even if SRS is not generally recommended for the primary treatment setting of bone metastases SRS represents a highly valuable treatment option in pre-irradiated patients.

## List of abbreviations

α/β: alpha/beta value; C: cervical vertebra; cc: cubic centimetre; CNS: central nervous system; CT: computed tomography; CTC: common toxicity criteria; GTV: gross tumour volume; Gy: Gray; IMRT: intensity modulated radiotherapy; L: lumbar vertebra; mets: metastases; MLC: multi-leaf collimator; MPNST: malignant peripheral nerve sheath tumour; MRI: magnetic resonance imaging; nBED: normalized biological effective dose; NSCLC: non-small-cell lung cancer; PFP: progression-free probability; PTV: planning target volume; QUANTEC: quantitative analyses of normal tissue effects in the clinic; RCC: renal call carcinoma; RECIST: response evaluation criteria in solid tumours; RTX: radiotherapy; S: sacral vertebra; SBRT: stereotactic body radiotherapy; SRS: spinal radiosurgery; T: thoracic vertebra; VAS: visual analogue scale.

## Conflict of interest statement

The authors declare that they have no competing interests.

## Authors' contributions

AM, BW and UG planned the study. KN, UG and MK collected and analysed the data, MK, KN and MN performed the statistics. Radiosurgery and clinical outcome data were collected by AM, BW and MK. KN prepared, KN and UG revised the manuscript. All authors read and approved the manuscript.
